# The Pharmacologically Active Alkaloid Cryptolepine Activates a Type 1 Interferon Response That Is Independent of MAVS and STING Pathways

**DOI:** 10.1155/2022/8873536

**Published:** 2022-07-26

**Authors:** Seth A. Domfeh, Patrick W. Narkwa, Osbourne Quaye, Kwadwo A. Kusi, Bright S. Addy, Sian Lant, Rebecca P. Sumner, Carlos Maluquer de Motes, Gordon A. Awandare, Charles Ansah, Mohamed Mutocheluh

**Affiliations:** ^1^West African Centre for Cell Biology of Infectious Pathogens, University of Ghana, Legon, Ghana; ^2^Department of Biochemistry, Cell and Molecular Biology, School of Biological Sciences University of Ghana, Legon, Ghana; ^3^Department of Biochemistry and Biotechnology, College of Science, Kwame Nkrumah University of Science and Technology, Kumasi, Ghana; ^4^Department of Clinical Microbiology, School of Medicine and Dentistry, Kwame Nkrumah University of Science and Technology, Kumasi, Ghana; ^5^Department of Immunology, Noguchi Memorial Institute for Medical Research, College of Health Sciences, University of Ghana, Legon, Ghana; ^6^Department of Microbial Sciences, University of Surrey, Guildford, UK; ^7^Department of Pharmacology, Faculty of Pharmacy and Pharmaceutical Sciences, College of Health Sciences, Kwame Nkrumah University of Science and Technology, Kumasi, Ghana

## Abstract

Type 1 interferons (IFN-1) are pleiotropic cytokines with well-established anticancer and antiviral properties, particularly in mucosal tissues. Hence, natural IFN-1-inducing treatments are highly sought after in the clinic. Here, we report for the first time that cryptolepine, a pharmacoactive alkaloid in the medicinal plant *Cryptolepis sanguinolenta*, is a potent IFN-1 pathway inducer. Cryptolepine increased the transcript levels of *JAK1*, *TYK2*, *STAT1*, *STAT2*, *IRF9*, and *OAS3*, as well as increased the accumulation of STAT1 and OAS3 proteins, similar to recombinant human IFN-*α*. Cryptolepine effects were observed in multiple cell types including a model of human macrophages. This response was maintained in MAVS and STING-deficient cell lines, suggesting that cryptolepine effects are not mediated by nucleic acids released upon nuclear or organelle damage. In agreement, cryptolepine did not affect cell viability in concentrations that triggered potent IFN-1 activation. In addition, we observed no differences in the presence of a pharmacological inhibitor of TBK1, a pleiotropic kinase that is a converging point for Toll-like receptors (TLRs) and nucleic acid sensors. Together, our results demonstrate that cryptolepine is a strong inducer of IFN-1 response and suggest that cryptolepine-based medications such as *C. sanguinolenta* extract could be potentially tested in resource-limited regions of the world for the management of chronic viral infections as well as cancers.

## 1. Introduction

Type 1 interferons are a family of pleiotropic cytokines and the main products of the innate immune response. IFN-1 are produced by several cells typically via the recognition of microbial molecular patterns by the cellular pattern recognition receptors (PRRs). Cytosolic RNA is normally sensed by the melanoma differentiation-associated gene 5 (MDA5) and retinoic acid-inducible gene 1 (RIG-1) which leads to the phosphorylation of TANK-bind kinase 1 (TBK1) through MAVS (mitochondrial antiviral-signalling protein) reviewed in [1]. Cytosolic DNA, on the other hand, is mostly sensed by the cyclic GMP-AMP synthase (cGAS) that generates the cyclic GMP-AMP (cGAMP) resulting in the activation of TBK1 through STING (stimulator of IFN genes protein) [2]. The activated TBK1 from these sensing pathways phosphorylates the IFN-regulatory factor 3 (IRF3) leading to the release of IFN-1 from the infected cells [3].

After their release, the IFN-1 interact with IFN-*α* receptors (IFNARs) on the infected and nearby cells resulting in the activation of the Janus kinase-signal transducer and activator of transcription (JAK-STAT) pathway. This causes the expression of numerous genes containing IFN-sensitive response elements (ISRE) in their promoter regions. These IFN-stimulated genes (ISGs) such as 2′5′-oligoadenylate synthase (OAS) and IFN-induced protein with tetratricopeptide repeats (IFIT), among others, are reviewed in [4]. These ISGs facilitate the antiviral and anticancer effects of IFN-1 reviewed in [5]. Moreover, the IFN-1 pathway exerts an anti-inflammatory response via the reduction in the levels of interleukin 1 (IL-1) and other inflammatory mediators [6]. Hence, impaired IFN-1 response has been reported to be allied to the increased susceptibility to viral infections and cancers [7].

IFN-1 are well-known immune mediators in the fight against viral infections, cancer, and autoimmune diseases and have a key role in immune protection in mucosal tissues [8, 9], the first line of defence against many pathogens. The integral role of IFN-1 in the mucosal barrier functions and immunity regulation has been demonstrated using animal models lacking IFN signalling, which fails to develop suitable immune responses to pathogenic microbes or their products [10]. The unique biological effects of endogenous IFN-1 have generated considerable interest in the use of recombinant IFNs in the treatment of cancers, viral infections, and immunological disorders [11, 12]. Although recombinant IFNs are effective, they have been reported with immunosuppressive effects [13] and are also associated with numerous adverse effects including fatigue, hepatotoxicity, retinal vascular complication, and biphasic thyroid dysfunction [14, 15]. Further, in low- and middle-income countries (LMICs), IFN-based therapies are hindered by the unavailability and high cost [16]. Owing to these limitations, there is a high interest in natural therapies that are less toxic, cheaper, and culturally acceptable [17].

Most people residing in LMICs use medicinal plants for their basic health care needs [18]. Moreover, these medicinal plants are easily accessible in these countries [19]. However, an important limitation in our ability to exploit these medicinal plants therapeutically is the absence of molecular studies revealing their immunomodulatory properties. Cryptolepine is the main biologically active alkaloid in the West African medicinal plant *Cryptolepis sanguinolenta* (Lindl.) Schlechter (Apocynaceae). This plant has been reported to exhibit a plethora of pharmacoeffects including antimalarial, antihyperglycaemic, antibacterial, and anti-inflammatory effects in diverse animal models reviewed in [20]. Owing to the convincing pharmacological properties of cryptolepine, *C. sanguinolenta* is used in Ghana for the treatment of malaria [21]. Although studies have investigated some of the pharmacological properties of cryptolepine [22–24], there is still scanty data on the immunomodulatory effects of cryptolepine specifically its effect on the IFN-1 response pathway.

Here, we established the mechanisms via which cryptolepine modulates the IFN-1 response pathway. We observed no differences in the IFN-1 response activation by cryptolepine in the absence of MAVS and STING, as well as impaired TBK1 signalling. Our findings provide evidence of the immune-boosting properties of *C. sanguinolenta*. This supports further studies to evaluate and potentially repurpose the beneficial effects of this plant for the treatment of chronic viral infections as well as cancers.

## 2. Materials and Methods

### 2.1. Chemicals and Reagents

The cryptolepine used in this study was prepared and stored as we have previously described [24]. It was isolated and purified from the roots of *C. sanguinolenta* by Professor Kwesi Boadu Mensah as described [25]. Recombinant Human Interferon Alpha 2b (IFN-*α*) (cat # 11105-1) was procured from PBL Assay Science (Piscataway, USA). HSV-60/LyoVec (cat # tlrl-hsv60c), 3′3 ′-cGAMP Fluorinated (cat # tlrl-nacgaf), and Poly(I:C)-HMW/LyoVec (cat # tlrl-piclv) were purchased from Invivogen (San Diego, USA). GSK8612 (cat # HY-111941) was purchased from MedChemExpress (New Jersey, USA). TransIT-2020 Transfection Reagent (cat # MIR 5400) was purchased from Mirus (Madison, USA). Dual Luciferase Reporter Assay System (cat # E1960) was procured from Promega (Madison, USA). Phorbol 12-myristate 13-acetate (cat # P1585), fludarabine (cat # F9813), and thiazolyl blue tetrazolium bromide (MTT) powder (cat # M5655-1G) were procured from Sigma-Aldrich (St. Louis, USA). Anti-STAT1 antibody (cat # ab210524), anti-OAS3 antibody (cat # ab154270), goat anti-rabbit IgG H&L (HRP) (cat # ab205718), and anti-beta actin antibody (cat # ab8227) were purchased from Abcam (Cambridge, USA). Anti-IFN-*α*/*β*R*α* antibody (cat # sc-7391) was procured from Santa Cruz Biotechnology (Texas, USA). Pierce enhanced chemiluminescence (ECL) western blotting substrate (cat # 32109), radioimmunoprecipitation assay (RIPA) buffer (cat # 89900), protease and phosphatase inhibitor cocktail (cat # 78440), ethylenediaminetetraacetic acid (cat # 78440), GeneJET RNA purification kit (cat # K0732), Maxima first-strand cDNA synthesis kit with dsDNase (cat # K1672), and ABsolute qPCR ROX mix (cat # AB1139A) were purchased from ThermoFisher Scientific (Massachusetts, USA). The chemicals and reagents were prepared in line with the manufacturers' instructions.

### 2.2. Cell Culture

The human monocytic leukaemia (THP1) cells stably expressing the interferon-induced protein with tetratricopeptide repeats 1- (IFIT1-) driven *Gaussia* luciferase gene (THP1-IFIT1-GLuc cells (parent cells), THP1-IFIT1-GLuc-MAVS−/− cells (MAVS KO cells), and THP1-IFIT1-GLuc-STING−/− cells (STING KO cells)) have been previously described [26–30]. How the various THP1 cells (parent and KO (knockout) cells) were modified and validated have been described previously [27, 29] (Supplementary data S3). Also, human hepatoma cells (HepG2, ATCC HB­8065), as well as human embryonic kidney cells (HEK293, ATCC CRL-1573), were bought from the American Type Culture Collection (ATCC, Manassas, USA). The HepG2 and HEK293 cells were used to buttress some of the results obtained using the THP1 cells (Supplementary data S1, S2, S4–S7). The THP1 cells were cultured in the RPMI­1640 Medium (ATCC) supplemented with 0.05 mM 2-*β*­mercaptoethanol (Bio-Rad, California, USA, cat # 1610710), 100 *μ*g/mL streptomycin and 100 IU/mL penicillin (Gibco Life Technologies Ltd., Paisley, UK, cat # 15140122), and 10% foetal bovine serum (FBS, ATCC 30-2020) to constitute the complete growth medium. However, the HepG2 and HEK293 cells were grown in Eagle's Minimum Essential Medium (EMEM, ATCC) containing 1% MEM nonessential amino acids (ScienCell, Carlsbad, USA, cat # 0823), 100 *μ*g/mL streptomycin, 100 IU/mL penicillin, and 10% FBS to make the complete growth medium. All the cells (THP1, HepG2, and HEK293) were cultured under a humidified condition at 37°C and in 5% carbon dioxide.

### 2.3. Cytotoxicity Assay

All the THP1 cells (parent, MAVS, and STING KO cells) were seeded each at 8 × 10^4^ cells per well in a 100 *μ*L complete growth medium containing 100 nM PMA (phorbol 12-myristate 13-acetate) in 96-well plates. At 72-hour postdifferentiation, the derived macrophages were treated with 0.5–4 *μ*M cryptolepine in a 100 *μ*L PMA-free complete growth medium. The viability of the cells was assessed after 24-hour posttreatment using an MTT-based assay as we have previously described [24].

### 2.4. Luciferase Reporter Gene Assay

All the THP1 cells (parent, MAVS, and STING KO cells) were seeded at 8 × 10^4^ cells per well in a 100 *μ*L complete growth medium containing 100 nM PMA in 96-well plates. At 72-hour postdifferentiation, the derived macrophages were cultured with cryptolepine alone or together with fludarabine (STAT1 inhibitor), GSK8612 (TBK1 inhibitor), anti-IFNAR1 blocking antibody, Poly(I:C) (an agonist of RIG-1/MDA5-MAVS axis), HSV-60 (an agonist of cGAS-STING axis), and IFN-*α* (an agonist of JAK-STAT axis) when necessary in 100 *μ*L PMA-free complete growth medium. After 24 hours of incubation, the *Gaussia* luciferase activity (which is the IFIT1 induction and the measure of the IFN-1 pathway response) was assessed in the presence of coelenterazine (2 *μ*g/mL) as previously done [30].

### 2.5. Reverse Transcriptase-Quantitative Polymerase Chain Reaction (RT-qPCR)

The parent THP1 cells were differentiated into macrophages at 2 × 10^6^ cells per well in a 6-well plate and cocultured in the presence of 60 *μ*M fludarabine and cryptolepine (2 and 4 *μ*M) or 400 IU/mL IFN-*α* (positive control). At 24-hour postculture, the total RNA was extracted with the GeneJET RNA Purification Kit in line with the manufacturer's protocols. The purity and yield of the extracted RNA were assessed by spectroscopy (NanoDrop 1000, Thermo Scientific), and the quality of the RNA was further validated by resolving on 1% agarose gel. The complementary DNA (cDNA) was synthesized using the Maxima first-strand cDNA synthesis kit with dsDNase in line with the manufacturer's instructions. The target genes (*JAK1*, *TYK2*, *STAT1*, *STAT1*, *IRF9*, and *OAS3*) were amplified using the ABsolute qPCR ROX mix with *β*-actin (*ACTB*) as an endogenous control. The primers and probes used in this study were synthesized by Biomers, Germany (Supplementary data S8). The amplification was done using the StepOnePlus Real-Time PCR System (Applied Biosystems, USA). After the initial holding for 10 minutes at 95°C, the PCR reaction was followed by 40 cycles for 15 seconds at 95°C and 60 seconds at 60°C. The relative quantification of the target genes was calculated using the comparative C_T_ method. The relative levels of the target genes after the normalisation to the endogenous gene (*ACTB*) were derived from the 2^-*ΔΔ*CT^ values as done previously [31].

### 2.6. Western Blotting

The protein levels of STAT1 and OAS3 were assessed using western blot to corroborate the expression of the mRNA levels. This is because the mRNA levels do not always translate into proteins [32, 33]. The protein level of *β*-actin (ACTB) was included as a loading control. The parent THP1 cells were differentiated into macrophages and treated as in the RT-qPCR. The total proteins were extracted using an ice-cold RIPA buffer which contains a protease and phosphatase inhibitor cocktail, as well as ethylenediaminetetraacetic acid, following the manufacturer's instructions. The extracted proteins were denatured in a sample buffer containing 2-beta mercaptoethanol. The denatured proteins were resolved using 4–20% gradient polyacrylamide gel and further transferred onto polyvinylidene difluoride (PVDF) membrane. The membrane was blocked in 5% nonfat dried milk in 1X Tris-buffered saline containing Tween-20 (TBST). The proteins of interest were probed using the primary specific antibodies: anti-STAT1 antibody (1 : 1000), anti-OAS3 antibody (1 : 1000), and anti-ACTB antibody (1 : 1000) in 3% nonfat dried milk in 1X TBST. The PVDF membrane was incubated with the primary antibodies for 18 hours at 4°C. The PVDF membrane was then probed with the goat anti-rabbit IgG H&L (HRP) secondary antibody (1 : 20000) at room temperature for 60 minutes. The PVDF membrane was incubated with the Pierce enhanced chemiluminescence western blotting substrate to visualise the bands. The images were taken using the C-DIGIT blot scanner (Li-COR Bioscience, USA) and further assessed using the ImageJ software (National Institute of Health).

### 2.7. Statistical Analysis

The data were analysed using Microsoft Excel (version 2019) where applicable. The viability of the cells was assessed as previously described [24]. The IFIT1 induction fold was derived from the ratio of IFIT1 induction (which is the IFN-1 pathway induction) in the cells cultured with cryptolepine or the ligands to the cells cultured without cryptolepine or the ligands [17]. Assessments among multiple treatments were done using the one-way analysis of variance (ANOVA) and Bonferroni's test for the post hoc analysis, whereas assessments between two treatments were conducted using Student's *t*-test. In all comparisons, a *p* value less than 0.05 was considered statistically significant.

## 3. Results

### 3.1. Cryptolepine Activates the IFN-1 Pathway in a Dose-Dependent Manner in Multiple Cells

**Figure 1:**
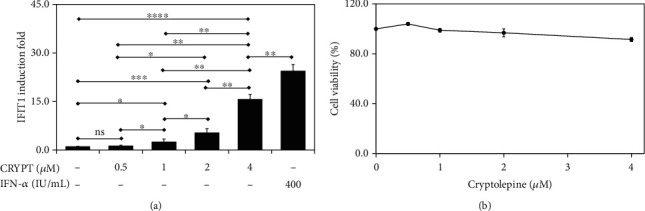
Cryptolepine activates IFN-1 response in THP1-derived macrophages. The derived macrophages from the parent THP1 cells were cultured with 0.5–4 *μ*M cryptolepine (CRYPT). IFN-*α* (400 IU/mL) was employed as an experimental positive control. The IFIT1 induction (a), as well as cell viability (b), was evaluated after 24 h. Data are shown as the means, from three varied experiments with each done in triplicate, and error bars represent the standard deviations. ^∗^*p* < 0.05, ^∗∗^*p* < 0.01, ^∗∗∗^*p* < 0.001, and ^∗∗∗∗^*p* < 0.0001; ns: difference not significant (one-way ANOVA and Bonferroni's test).

To study the ability of cryptolepine to induce IFN-1 responses, we initially used differentiated human THP-1 monocytes expressing Gaussia luciferase (GLuc) under the control of the promoter of the IRF3- and ISRE-dependent IFIT1 gene [26]. Cryptolepine at 0.5–4 *μ*M increased the IFIT1 induction (which is a measure of the IFN-1 pathway response) in a dose-dependent manner and with at least two-fold induction in the presence of 1 *μ*M cryptolepine in the derived macrophages (Figure 1(a)). Recombinant human IFN-*α* 2b (hereafter referred to as IFN-*α*, JAK-STAT agonist) was included in the experiment as a positive control. Moreover, the concentrations of cryptolepine used in this experiment (0.5–4 *μ*M) were not toxic to the differentiated macrophages since the viability of the cells remained > 80% after 24 hours (Figure 1(b)). To determine whether the observed effects were unique to the human macrophage model used, we tested HepG2 and HEK293 cells. Similar patterns of IFN-1 pathway induction by cryptolepine were observed in these cell lines (Supplementary data S1), suggesting that the cryptolepine mechanism of action is not cell type specific.

### 3.2. Cryptolepine Increases the mRNA Levels of *JAK1*, *TYK2*, *STAT1*, *STAT2*, *IRF9*, and *OAS3*

**Figure 2:**
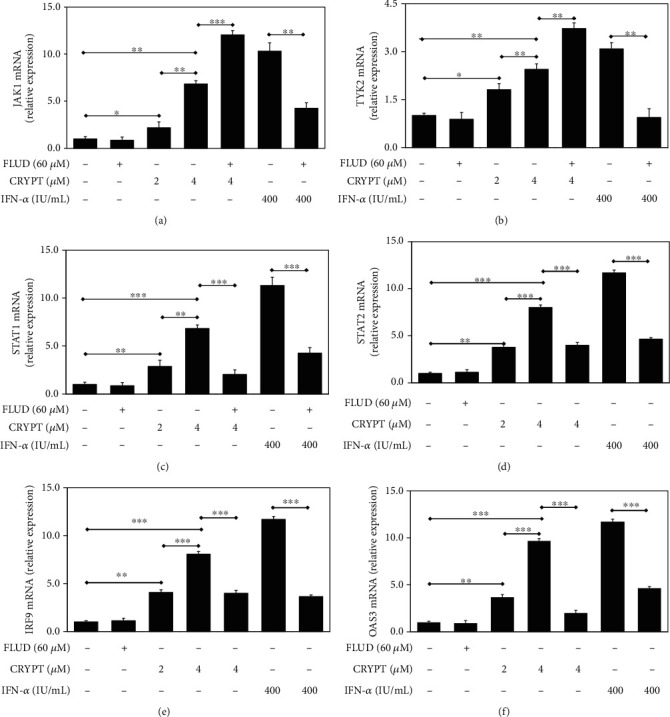
Cryptolepine increases *JAK1*, *TYK2*, *STAT1*, *STAT2*, *IRF9*, and *OAS3* mRNA levels in THP1-derived macrophages. The derived macrophages from the parent THP1 cells were cultured with cryptolepine (CRYPT), fludarabine (FLUD), or IFN-*α* as indicated. The target genes were assessed after 24 h by RT-qPCR with gene-specific primers and probes using *β*-actin as an endogenous control. Data are shown as the means, from three varied experiments with each done in triplicate, and error bars represent the standard deviations. ^∗^*p* < 0.05, ^∗∗^*p* < 0.01, and ^∗∗∗^*p* < 0.001 (one-way ANOVA and Bonferroni's test).

To confirm the effects of cryptolepine on the IFN-1 pathway, we assessed endogenous gene expression by RT-qPCR. Cryptolepine increased the expressions of key transcripts of the IFN-1 response pathway such as *JAK1*, *TYK2*, *STAT1*, *STAT2*, and *IRF9*, as well as *OAS3* (a well-established ISG), in a dose-dependent manner (Figure 2). A similar increase in mRNA levels was observed in HepG2 cells (Supplementary data S2). Except for *JAK1* and *TYK2*, the elevated mRNA levels induced by cryptolepine were reduced in the presence of fludarabine, a pharmacological inhibitor of STAT1, suggesting that cryptolepine effects were STAT1 dependent.

### 3.3. Cryptolepine Increases the Protein Levels of STAT1 and OAS3 in THP1-Derived Macrophages

**Figure 3:**
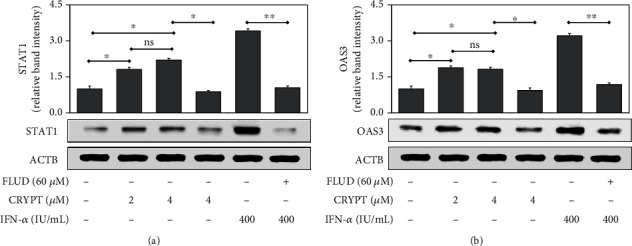
Cryptolepine increases STAT1 and OAS3 protein levels in THP1-derived macrophages. The derived macrophages were cotreated with fludarabine (FLUD) and cryptolepine (CRYPT) or IFN-*α*. The target proteins were assessed after 24 h by western blotting using the indicated antibodies. *β*-Actin (ACTB) was included as a protein loading control. The images are representative of three varied experiments. ^∗^*p* < 0.05 and ^∗∗^*p* < 0.01; ns: difference not significant (one-way ANOVA and Bonferroni's test).

We then assessed whether the transcriptional changes resulted in increased protein levels. Cryptolepine increased the protein levels of STAT1 (Figure 3(a)) and OAS3 (Figure 3(b)) in the derived macrophages. In agreement with the previous results (Figures 2(c) and 2(f)), the elevated STAT1 and OAS3 levels were reversed in the presence of fludarabine similar to the IFN-*α*-positive control.

### 3.4. IFN-1 Pathway Activation in the Presence of Cryptolepine Does Not Require MAVS, STING, and TBK1 in THP1-Derived Macrophages

**Figure 4:**
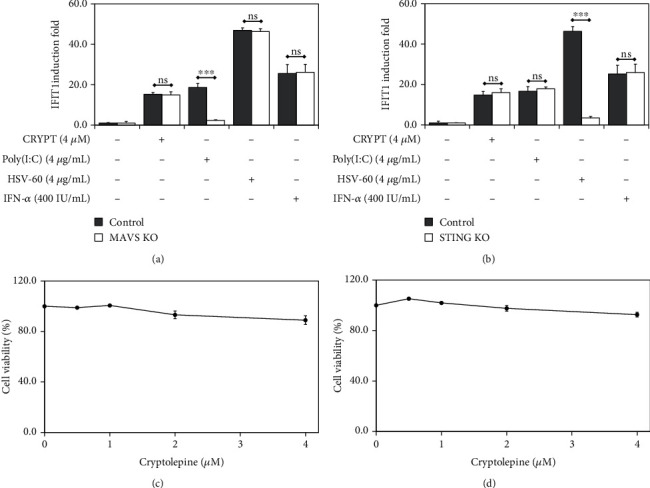
Cryptopine activates IFN-1 response in MAVS and STING-deficient THP1-derived macrophages. The derived macrophages from the MAVS (a) and STING (b) deficient THP1 cells were cultured with cryptolepine (CRYPT), Poly(I:C), HSV-60, or IFN-*α*. The derived macrophages from the parent THP1 cells served as the control to which the IFIT1 induction in the deficient cells was compared. Also, the cytotoxic effects of cryptolepine on the MAVS (c) and STING (d) deficient cells were assessed. Data are shown as the means, from three varied experiments with each done in triplicate, and error bars represent the standard deviations. ^∗∗∗^*p* < 0.001; ns: difference not significant (Student's *t*-test).

IFN-1 are typically produced by activation of PRRs such as TLRs or cytosolic receptors. Engagement of IFN-1 with their cognate receptor results in activation of the IFN-1 pathway. To establish whether the effects of cryptolepine on the IFN-1 pathway were mediated by cytosolic nucleic acid sensors, we tested cryptolepine in THP-1 cells unable to respond to cytosolic RNA or DNA due to the absence of the main RNA and DNA sensors MAVS and STING (Supplementary data S3 and [27–29]). IFIT1 induction by cryptolepine was indistinguishable in the macrophages derived from the MAVS and STING-deficient cells as compared to the parent cells (Figures 4(a) and 4(b)). As expected, MAVS-deficient cells were unable to respond to Poly(I:C) (a synthetic dsRNA analogue) (Figure 4(a)), and STING-deficient cells were unable to respond to HSV-60 (a DNA motif derived from herpes simplex virus-1 genome) (Figure 4(b)). These results indicate that cryptolepine activates the IFN-1 response independent of MAVS and STING signalling, suggesting that cryptolepine does not trigger intracellular damage resulting in the leakage of nucleic acids from organelles or the nuclear envelope. This was in line with the absence of noticeable toxicity in the deficient cells exposed to cryptolepine (Figures 4(c) and 4(d)) similar to the parental cells (Figure 1(b)).

**Figure 5:**
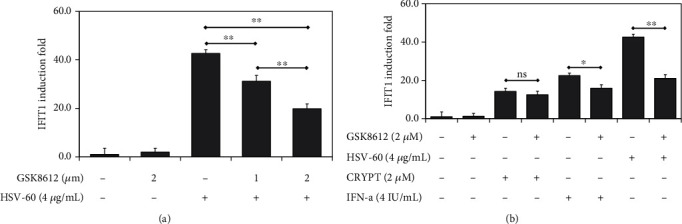
Cryptolepine activates IFN-1 response regardless of TBK1 inhibition in THP1-derived macrophages. The derived macrophages from the parent THP1 cells were cocultured with GSK6812 and HSV-60 (a) or GSK6812 and cryptolepine (CRYPT) (b), and the IFIT1 induction was evaluated after 24 h. Data are shown as the means, from three varied experiments with each done in triplicate, and error bars represent the standard deviations. ^∗^*p* < 0.05, ^∗∗^*p* < 0.01, and ^∗∗∗^*p* < 0.001; ns: difference not significant (one-way ANOVA and Bonferroni's test (a) or Student's *t*-test (b)).

Upon recognition of RNA and DNA by cytosolic nucleic acid sensors, adaptors MAVS and STING mediate activation of the kinase TBK1. TBK1 is also a converging point for IFN-1 activation mediated by TLRs. To establish whether the effects of cryptolepine on the IFN-1 pathway were dependent on TBK1 activation, we tested cryptolepine in the presence of the TBK1 inhibitor GSK8612. TBK1 inhibition in the macrophages derived from the parent THP1 cells was first established using the HSV-60. There was a dose-dependent suppression of the IFIT1 induction by GSK8612 in the presence of HSV-60 (Figure 5(a)). The GSK8612 at 2 *μ*M was used to inhibit the TBK1 in the derived macrophages because this concentration suppressed over 50% of the IFIT1 induction. Further, the IFIT1 induction by cryptolepine was unaffected by GSK8612 (Figure 5(b)). These results imply that cryptolepine activates the IFN-1 response independent of TBK1 signalling. Also, similar patterns of results as observed in the derived macrophages were observed in HepG2 and HEK293 cells (Supplementary data S4).

### 3.5. IFN-1 Pathway Activation in the Presence of Cryptolepine Requires IFNAR1 and STAT1

**Figure 6:**
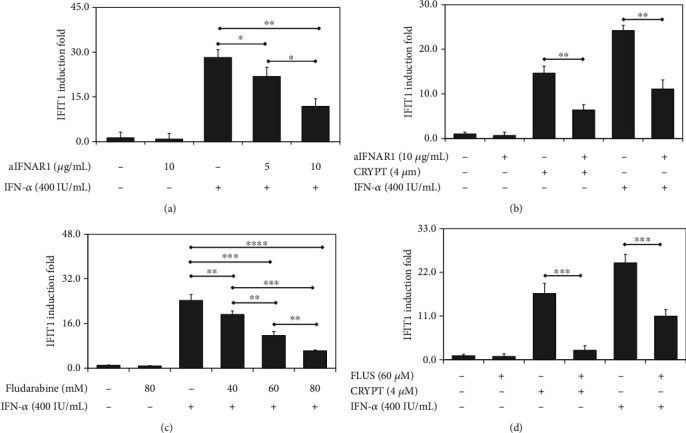
Cryptolepine activates IFN-1 response through IFNAR1 and STAT1 in THP1-derived macrophages. The derived macrophages from the parent THP1 cells were cocultured with the anti-IFNAR1 blocking antibody (aIFNAR1) and IFN-*α* (a) or cryptolepine (CRYPT) (b), and fludarabine (FLUD) and IFN-*α* (c) or cryptolepine (d) and the IFIT1 induction was evaluated after 24 h. Data are shown as the means, from three varied experiments with each done in triplicate, and error bars represent the standard deviations. ^∗^*p* < 0.05, ^∗∗^*p* < 0.01, ^∗∗∗^*p* < 0.001, and ^∗∗∗∗^*p* < 0.0001 (one-way ANOVA and Bonferroni's test (a and c) or Student's *t*-test (b and d)).

We then gained more mechanistic insights using IFNAR antibody blockade and STAT1 inhibitor. We first determined a dose-dependent suppression of the IFIT1 induction in the presence of the anti-IFNAR1 blocking antibody and IFN-*α* (Figure 6(a)). The anti-IFNAR1 blocking antibody at 10 *μ*g/mL suppressed over 50% of the IFIT1 induction and was used to block IFNAR1 in further experiments. Further, when the IFNAR1 was blocked in the derived macrophages from the parent THP1 cells, the IFIT1induction by cryptolepine was over 50% suppressed (Figure 6(b)). Similar patterns of results as observed in the derived macrophages were observed in HepG2 and HEK293 cells (Supplementary data S5). Further, the effect of STAT1 inhibition on the IFIT1 induction by cryptolepine was assessed. There was a dose-dependent suppression of the IFIT1 induction by fludarabine in the presence of IFN-*α* (Figure 6(c)). Fludarabine at 60 *μ*M was used to inhibit STAT1 because this concentration suppressed over 50% of the IFIT1 induction. Next, when STAT1 was inhibited in the derived macrophages from the parent THP1 cells, the IFIT1 induction by cryptolepine was over 80% suppressed (Figure 6(d)). Also, similar patterns of results as observed in the derived macrophages were observed in HepG2 and HEK293 cells (Supplementary data S6). Taken together, these results demonstrate that cryptolepine activates the IFN-1 pathway via IFNAR1 and STAT1.

### 3.6. Cryptolepine Enhances IFN-1 Pathway Activation by Poly(I:C), HSV-60, and IFN-*α*

**Figure 7:**
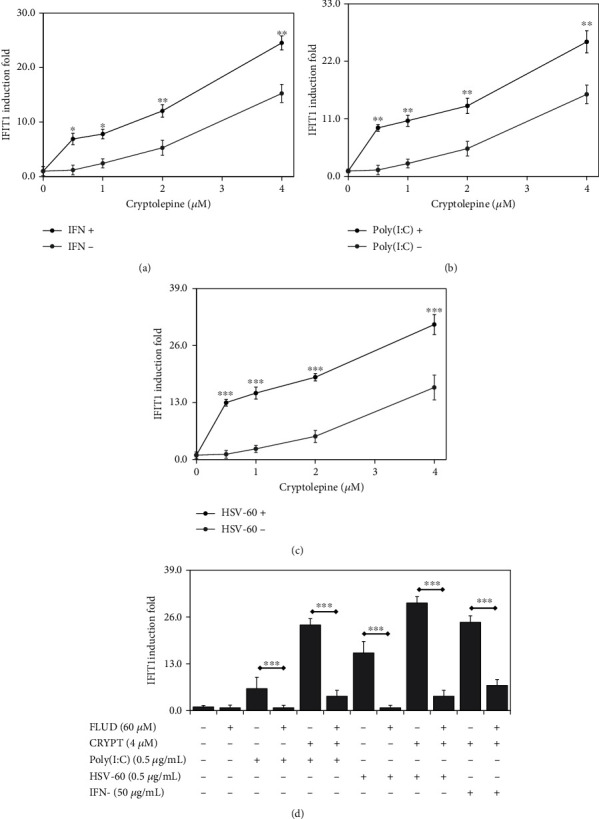
Cryptolepine enhances IFN-1 response activation by Poly(I:C), HSV-60, and IFN-*α* in THP1-derived macrophages. The derived macrophages from the parent THP1 cells were cultured with cryptolepine and 50 IU/mL IFN-*α* (a), 0.5 *μ*g/mL Poly(I:C) (b), and 0.5 *μ*g/mL HSV-60 (c), and the IFIT1 induction was assessed after 24 h. Moreover, the macrophages were cocultured with fludarabine (FLUD), IFN-*α* (Poly(I:C) or HSV-60), and cryptolepine, and the IFIT1 induction was evaluated after 24 h (d). Data are shown as the means, from three varied experiments with each done in triplicate, and error bars represent the standard deviations. ^∗^*p* < 0.05, ^∗∗^*p* < 0.01, ^∗∗∗^*p* < 0.001, and ^∗∗∗∗^*p* < 0.0001 (Student's *t*-test).

To determine whether cryptolepine effects could negatively or positively impact responses induced by innate immune agonists, we cultured differentiated macrophages in the presence of cryptolepine together with IFN-*α*, Poly(I:C), or HSV-60 (Figures 7(a)–7(c), respectively). Cryptolepine enhanced IFIT1 induction in all conditions tested, and this was suppressed when STAT1 was inhibited (Figure 7(d)). These data together indicate that cryptolepine-mediated IFN-1 activation synergises with activation induced by other agonists and suggest that, during viral infection, the IFN-1 response will be amplified in the presence of cryptolepine.

## 4. Discussion

IFN-1 play a significant function in the innate immune response as the first line of defence against pathogens. Before infections, the exposure of cells to IFN-1 has been reported to induce an antiviral state that subsequently averts productive viral infection [34, 35]. Also, the IFN-1 have been reported to be hepatoprotective through the induction of the anti-inflammatory IL-1 receptor antagonist [36]. Further, the IFN-1 play a key role in immune protection in mucosal tissues leading to the restriction of enteric virus infections [37–39]. Moreover, IFN-1 have anticancer effects against non-Hodgkin lymphoma, chronic myeloid leukaemia, and hairy cell leukaemia [40]. With these beneficial properties, recombinant IFN-1 have been used in the management of viral infections, cancers, and immunological disorders [11, 12].

Medicinal plants are emerging as sources of compounds with notable pharmacological effects reviewed in [41]. One such compound that needs to be investigated is cryptolepine, which is the major pharmacoactive alkaloid in *C. sanguinolenta*. The aqueous root extract of *C. sanguinolenta* is being used for years as a tonic that is often taken daily with no reported toxicity and has hence been used in Ghana for the management of malaria [21]. Besides its plasmodiocidal activity, this medicinal plant has numerous pharmacological effects including, but not limited to, anticancer, antipyretic, anti-inflammatory, antithrombotic, antidiabetic, antiprotozoal, antifungal, antibacterial, and antihypertensive effects reviewed in [42]. Studies have shown that these pharmacological effects of *C. sanguinolenta* are attributed to mainly the presence of its main alkaloid cryptolepine [22, 24, 43–45].

**Figure 8:**
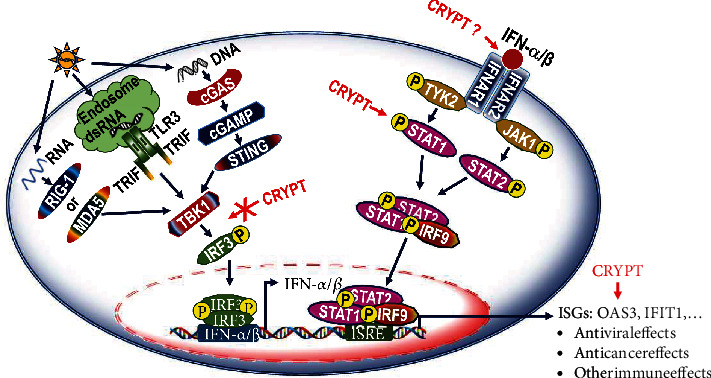
Proposed upregulation of the IFN-1 response pathway by cryptolepine. Cytosolic RNA is detected by the MDA5 or RIG-1 which leads to the activation of IRF3 via the TBK1. However, cytosolic DNA is recognised by cGAS which then stimulates the STING-dependent signalling, leading to the phosphorylation of IRF3. The phosphorylated IRF3 homodimerises and moves into the nucleus causing the release of the IFN-1 (mainly IFN-*α*/*β*). The secreted IFNs simulate the later phase of the IFN-1 pathway (JAK-STAT axis) through the IFNARs, which transmit a cascade of signalling events to activate the promoters of ISGF3 and induce the production of numerous IFN-responsive proteins which facilitate anticancer and antiviral responses. This study has demonstrated that cryptolepine activates the IFN-1 response pathway via the JAK-STAT axis (as indicated by arrows) but is independent of the first phase of the pathway (as indicated by a crossed arrow). CRYPT: cryptolepine.

Cryptolepine activated IFN-1 response similar to recombinant human IFN-*α* which supports the beneficial properties of cryptolepine similar to IFN-1. The IFN-1 response pathway is known to be hepatoprotective and antiviral [36, 46], and therefore, it could be deduced that cryptolepine has hepatoprotective properties, and this supports the ethnomedical use of *C. sanguinolenta* for the suggested management of viral hepatitis in Ghana. Moreover, OAS3 was reported to restrict intracellular replication of *Mycobacterium tuberculosis* [47]. Therefore, the increase in levels of OAS3 protein in the presence of cryptolepine (Figure 8) supports the ethnopharmacological use of *C. sanguinolenta* in the management of tuberculosis [48]. Further, the IFN-1 response is activated during malaria which leads to parasite clearance [49, 50]. This could explain the mechanism through which cryptolepine exhibits its antiplasmodial effect and the ethnomedical use of *C. sanguinolenta* for the treatment of malaria.

The anticancer role of STAT1 is considered to be important during the onset of tumourigenesis where it promotes the destruction of transformed cells by the immune system [51]. Moreover, STAT1 signalling has been reported to impede the proliferation of hepatocellular carcinoma through the induction of p53 signalling [52]. Hence, the increase in levels of STAT1 protein in the presence of cryptolepine (Figure 8) gives credence to its anticancer properties [23, 24, 43].

During mitochondrial stress, there is the leakage of mitochondrial (mt) DNA into the cytoplasm [53]. The mtDNA has been shown to trigger the cGAS-STING signalling and IFN-1 production [54, 55]. Hence, the activation of IFN-1 response by cryptolepine independent of STING implies that cryptolepine is not causing substantial cellular and mitochondrial stress. This is supported by the absence of noticeable toxicity in the cells exposed to cryptolepine concentrations used to activate the IFN-1 response pathway. Some cancer cells, including ovarian and liver cancer cells, exhibit impaired STING signalling [56, 57]. Also, defective STING signalling in human osteosarcoma cells has been reported to contribute to the growth of HSV-1 [58]. Further, MAVS dimer although crucial in innate immunity is a target for HCV NS3/4A protease [59]. Moreover, a heterozygous mutation in TBK1 impairs TLR3 signalling [60]. Therefore, the activation of IFN-1 response by cryptolepine regardless of the STING, MAVS, and TBK1 could be clinically beneficial.

## 5. Conclusion

Taken together, we have shown for the first time that cryptolepine is a potent IFN-1 response inducer. Additionally, we have revealed some antimicrobial and immune-boosting mechanistic activities of cryptolepine. Since cryptolepine has similar beneficial effects as IFN-1, we recommend that further studies should be conducted to potentially repurpose *C. sanguinolenta* preparation as a natural and cheaper immunotherapeutic drug for the management of chronic viral infections as well as cancers in resource-limited settings of the world.

## 6. Limitations

Although the results from this *in vitro* study are encouraging, there were some limitations. The experiments were not performed in primary cells and *in vivo*. Also, the effects of cryptolepine on IRF3 phosphorylation, IFN-*α*, and IFN-*β* levels were not assessed due to financial constraints. Further, the actual interaction between cryptolepine and the IFNARs was not assessed. Hence, we recommend that further studies should be conducted in primary cells and *in vivo* as well as ascertain the effects of cryptolepine on type 2 and 3 IFN pathways.

## Data Availability

The datasets used during the current study are available from the corresponding author on reasonable request.

## Abbreviations

AMP: Adenosine monophosphate
cGAMP: Cyclic guanosine monophosphate-adenosine monophosphate
cGAS: Cyclic GMP-AMP synthase
GMP: Guanosine monophosphate
HBV: Hepatitis B virus
HCC: Hepatocellular carcinoma
HCV: Hepatitis C virus
HRP: Horseradish peroxidase
IFIT: Interferon-induced protein with tetratricopeptide repeats
IFN: Interferon
IFNAR: Interferon alpha receptor
ISRE: Interferon stimulated response elements
JAK: Janus kinase
LMICs: Low- and middle-income countries
Luc: Luciferase
MAVS: Mitochondrial antiviral sensing protein
MDA5: Melanoma differentiation-associated gene 5
OAS: Oligoadenylate synthase
PMA: Phorbol 12-myristate 13-acetate
RIG-1: Retinoic acid-inducible gene 1
STAT: Signal transducer and activator of transcription
STING: Stimulator of interferon genes
TANK: TRAF family member-associated NF*κ*B activator
TBK: TANK-binding kinase

## Supplementary Materials

Supplementary Materials The supplementary material file contains the data obtained from the HepG2 and HEK293 cells (Supplementary data S1, S2, S4 – S7) as well as the validation of the MAVS and STING THP1-deficient cells (Supplementary data S 3). Also, the file contains the primers used in the application of the gene of interest (Supplementary data S8).
