# Supplementary material for: The Pharmacologically Active Alkaloid Cryptolepine Activates a Type 1 Interferon Response That Is Independent of MAVS and STING Pathways
Source: J Immunol Res. 2022 Jul 26;2022:8873536. doi: 10.1155/2022/8873536 (PMC9345703; doi:10.1155/2022/8873536)
Supplement: Supplementary Materials — The supplementary material file contains the data obtained from the HepG2 and HEK293 cells (Supplementary data S1, S2, S4 – S7) as well as the validation of the MAVS and STING THP1-deficient cells (Supplementary data S 3). Also, the file contains the primers used in the application of the gene of interest (Supplementary data S8). [file 8873536.f1.pdf]

The pharmacologically active alkaloid Cryptolepine activates a type 1 interferon response that is independent of MAVS and STING pathways

Seth A. Domfeh[1,2,5], Patrick W. Narkwa[3], Osbourne Quaye[1,2], Kwadwo A. Kusi[1,2,4], Bright S. Addy[5], Sian Lant[6], Rebecca P. Sumner[6], Carlos Maluquer de Motes[6], Gordon A. Awandare[1,2], Charles Ansah[7] and Mohamed Mutocheluh[3]*

[1]West African Centre for Cell Biology of Infectious Pathogens, University of Ghana, Legon, Ghana.

[2]Department of Biochemistry, Cell and Molecular Biology, School of Biological Sciences University of Ghana, Legon, Ghana.

[3]Department of Clinical Microbiology, School of Medicine and Dentistry, Kwame Nkrumah University of Science and Technology, Kumasi, Ghana.

[4]Department of Immunology, Noguchi Memorial Institute for Medical Research, College of Health Sciences, University of Ghana, Legon, Ghana.

[5]Department of Biochemistry and Biotechnology, College of Science, Kwame Nkrumah University of Science and Technology, Kumasi, Ghana.

[6]Department of Microbial Sciences, University of Surrey, Guildford, United Kingdom

[7]Department of Pharmacology, Faculty of Pharmacy and Pharmaceutical Sciences, College of Health Sciences, Kwame Nkrumah University of Science and Technology, Kumasi, Ghana.

*Corresponding author: mmutocheluh.chs@knust.edu.gh or mutocheluh@gmail.com

Supplementary Data

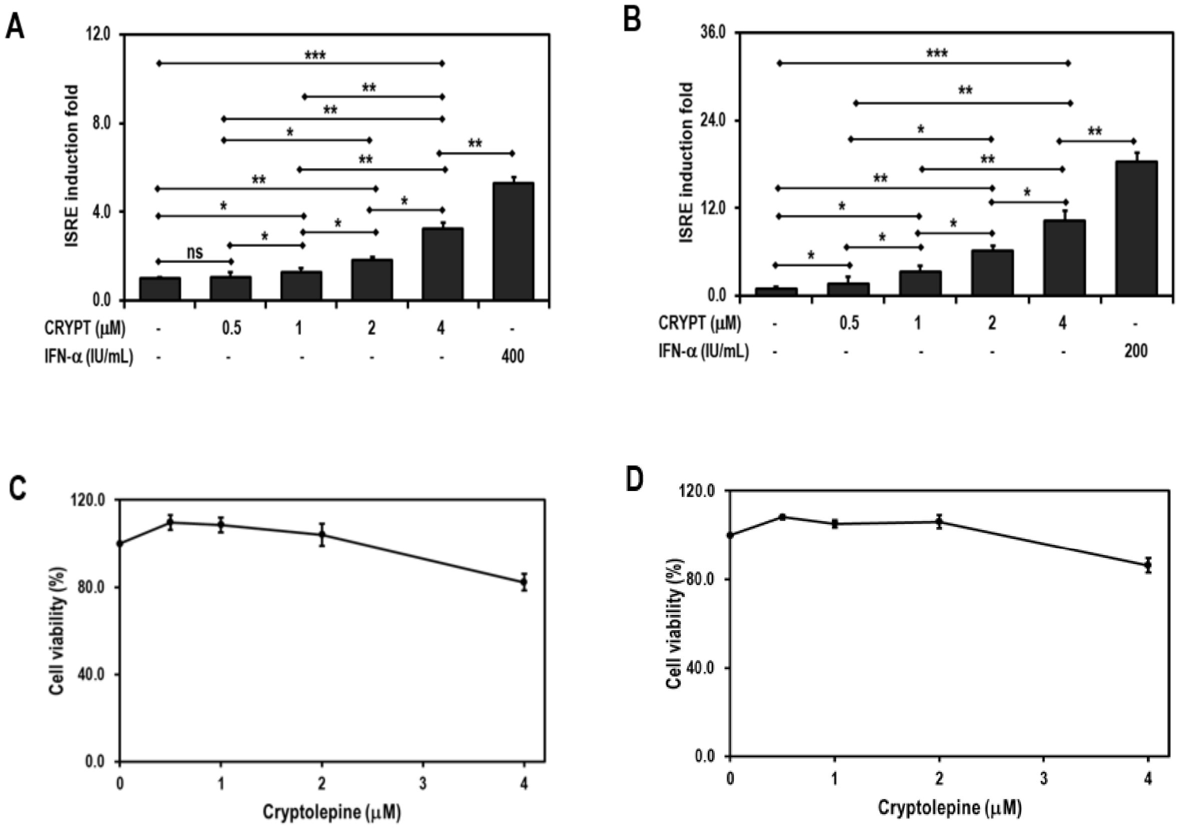

S 1: Cryptolepine activates IFN-1 response in HepG2 and HEK293 cells. The HepG2 (**A** and **C**) and HEK293 (**B** and **D**) cells were cultured at 4×10^4 cells per well in a 96-well plate. After 24 h, the cells were transiently co-transfected with pISRE-Luc (100 ng per well) and pRLSV40 (5 ng per well). The pRLSV40 which constitutively expresses the *Renilla* luciferase was included as an internal control to which the pISRE-Luc induction was normalised. At 24 h post-transfection, the cells were cultured with cryptolepine or IFN-α (positive control), and the ISRE induction (which is a measure of the IFN-1 pathway response), as well as cell viability, was assessed after 24 h. Data are presented as the means, with the standard deviations as error bars, from three different experiments and each conducted in triplicate. The concentrations of cryptolepine used in this experiment (0.5 – 4 μM) were non-toxic to the cells with a viability of over 80% after 24 hours. *$p < 0.05$, **$p < 0.01$, ***$p < 0.001$ and **ns**: difference not significant (one-way ANOVA and Bonferroni's test).

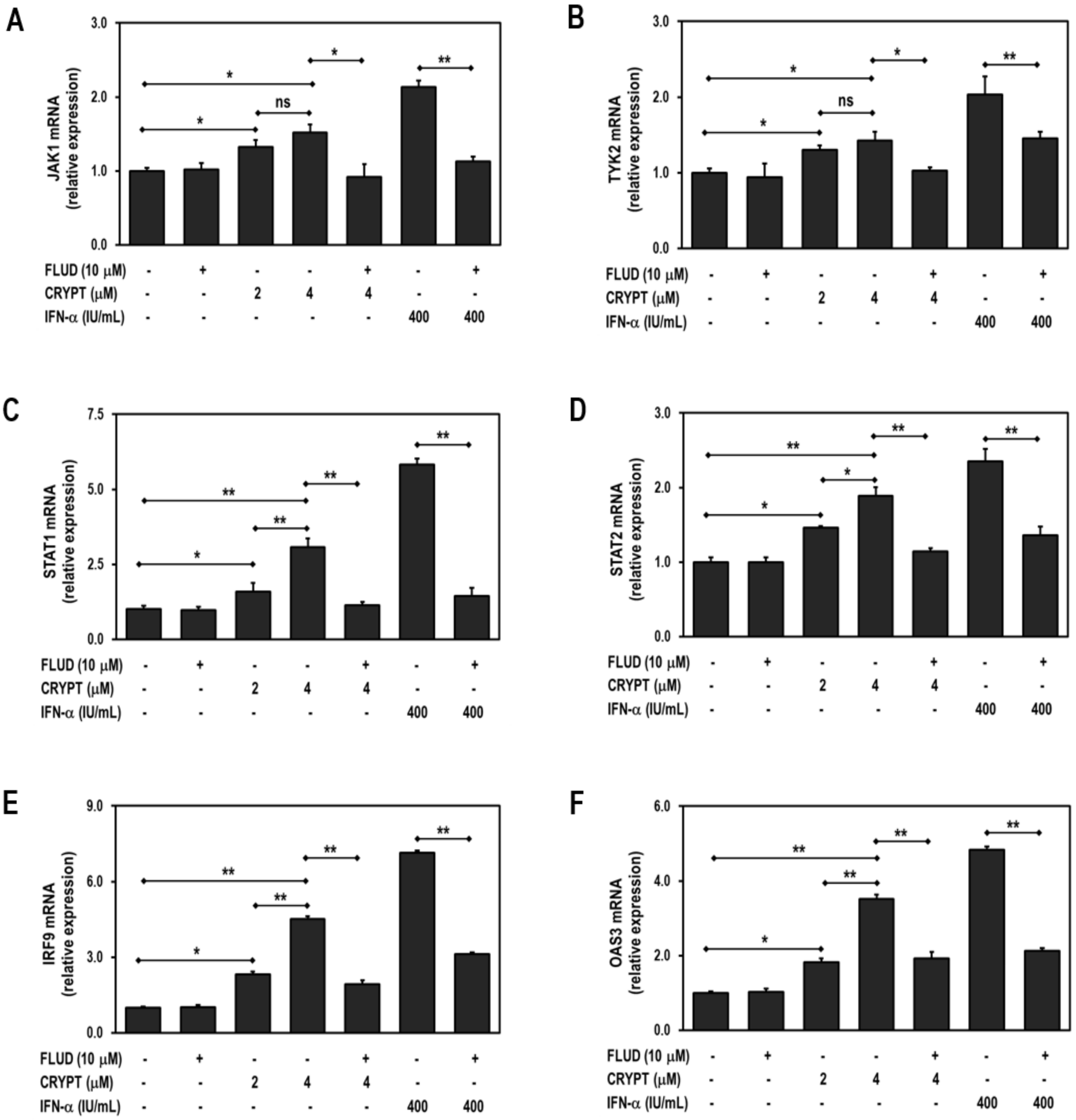

S 2: Cryptolepine increases *JAK1, TYK2, STAT1, STAT2, IRF9* and *OAS3* mRNA levels in HepG2 cells. The cells were cultured at 1.5 × 10^6 cells per well in a 6-well plate for 24 h. The cells were then pre-cultured with fludarabine (FLUD) and further cultured with cryptolepine (CRYPT) or IFN-α (positive control) in fludarabine-free media. The total RNA was extracted after 24 h and the target genes were assessed by RT-qPCR with genes specific primers and probes (**S 8**). Data are presented as the means, with the standard deviations as error bars, from three different experiments and each conducted in triplicate. *$p < 0.05$, **$p < 0.01$ and **ns**: difference not significant (one-way ANOVA and Bonferroni's test).

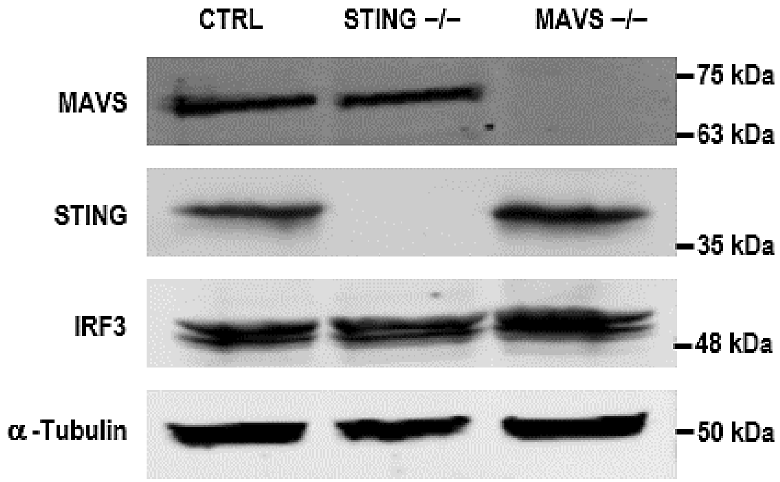

S 3: Immunoblot of THP1 cells deficient for STING or MAVS. The cells were lysed in RIPA buffer supplemented with protease and phosphatase inhibitors. Lysates were incubated at 4 °C for 30 mins with intermittent mixing and subsequently denatured for 5 min at 95°C in the presence of loading buffer. Samples were resolved by SDS-PAGE and then transferred to nitrocellulose membranes. The membranes were blocked using 0.1% Tween/phosphate-buffered saline supplemented with 5% skimmed milk and further incubated with the protein-specific primary antibodies. Primary antibodies were then detected using IRDye-conjugated secondary antibodies in an Odyssey infrared imaging system. Images were analysed using the Odyssey software. CTRL: Parent THP1 cells. This immunoblot experiment was performed by Sian Lant and Rebecca P. Sumner in Dr Carlos Maluquer de Motes' Laboratory, University of Surrey, UK. The THP1 cells deficient for STING or MAVS have been previously described (Gutierrez-Merino et al., 2020[1]; Hernaez et al., 2020[2]).

[1] Gutierrez-Merino, J., Isla, B., Combes, T., Martinez-Estrada, F., & Maluquer De Motes, C. (2020). Beneficial bacteria activate type-I interferon production via the intracellular cytosolic sensors STING and MAVS. *Gut Microbes*, 1-18.

[2] Hernáez, B., Alonso, G., Georgana, I., El-Jesr, M., Martín, R., Shair, K. H., . . . Alcamí, A. (2020). Viral cGAMP nuclease reveals the essential role of DNA sensing in protection against acute lethal virus infection. *Science advances*, *6*(38), 4565-4578.

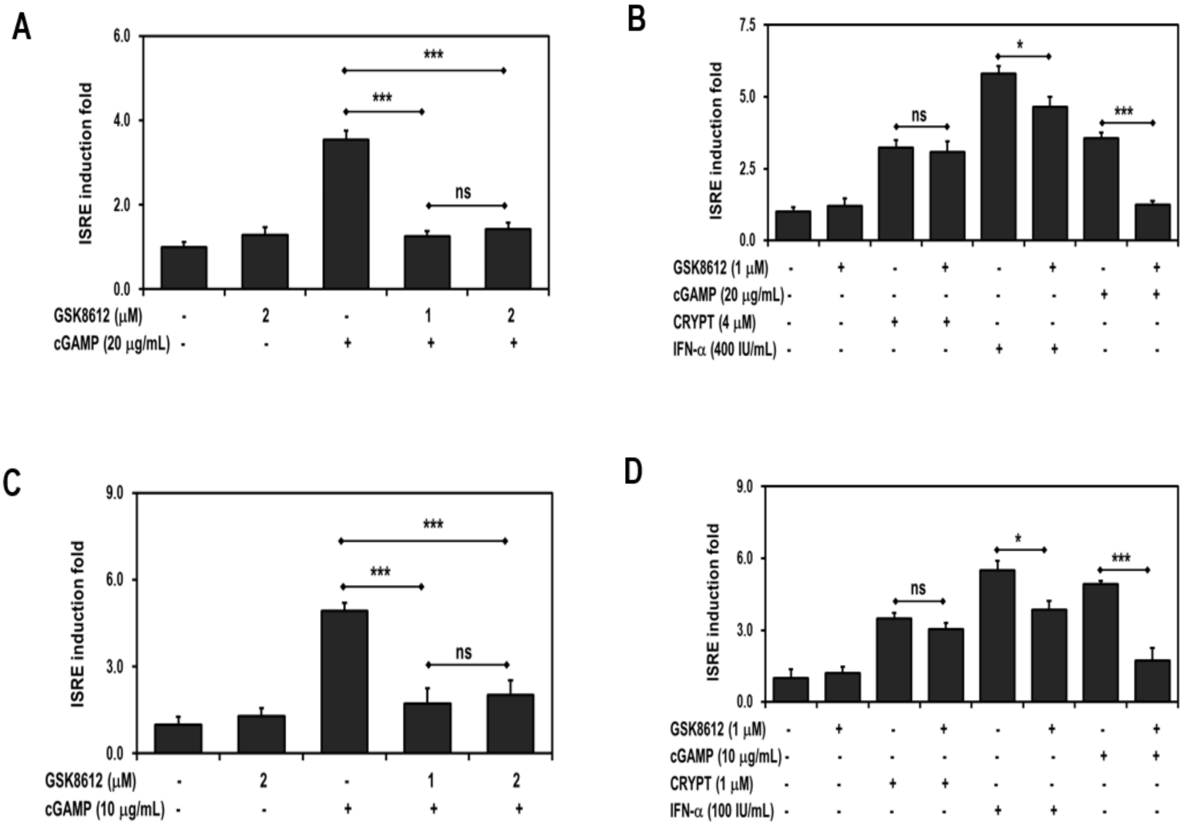

S 4: Cryptolepine activates IFN-1 response regardless of TBK1 inhibition in HepG2 and HEK293 cells. The HepG2 (**A** and **B**) and HEK293 (**C** and **D**) cells were cultured at $4{\times}10^4$ cells per well in a 96-well plate. After 24 h, the cells were transiently co-transfected as described under **S 1**. At 24 h post-transfection, cells were co-cultured with GSK6812 and cGAMP or cryptolepine (CRYPT) and the ISRE induction was assessed after 24 h. Data are presented as the means, with the standard deviations as error bars, from three different experiments and each conducted in triplicate. *$p < 0.05$, ***$p < 0.001$ and **ns**: difference not significant (one-way ANOVA and Bonferroni's test; **A** and **C** or student's *t*-test; **B** and **D**).

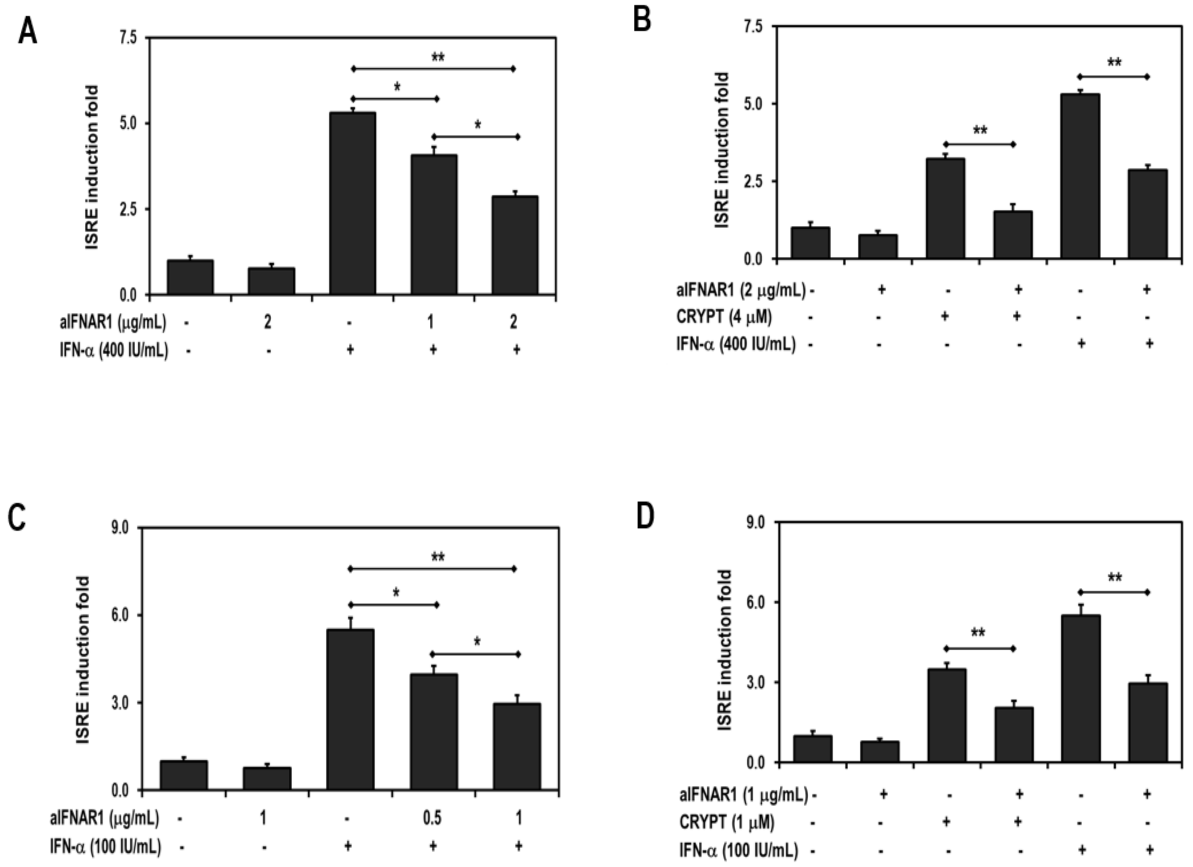

S 5: Activation of IFN-1 response by cryptolepine requires IFNAR1 in HepG2 and HEK293 cells. The HepG2 (**A** and **B**) and HEK293 (**C** and **D**) cells were cultured at $4×10^4$ cells per well in a 96-well plate. After 24 h, the cells were transiently co-transfected as described under **S 1**. At 24 h post-transfection, cells were co-cultured with anti-IFNAR1 blocking antibody (aIFNAR1) and IFN-α or cryptolepine (CRYPT) and the ISRE induction was assessed after 24 h. Data are presented as the means, with the standard deviations as error bars, from three different experiments and each conducted in triplicate. $*p < 0.05$ and $**p < 0.01$ (one-way ANOVA and Bonferroni's test; **A** and **C** or student's *t*-test; **B** and **D**).

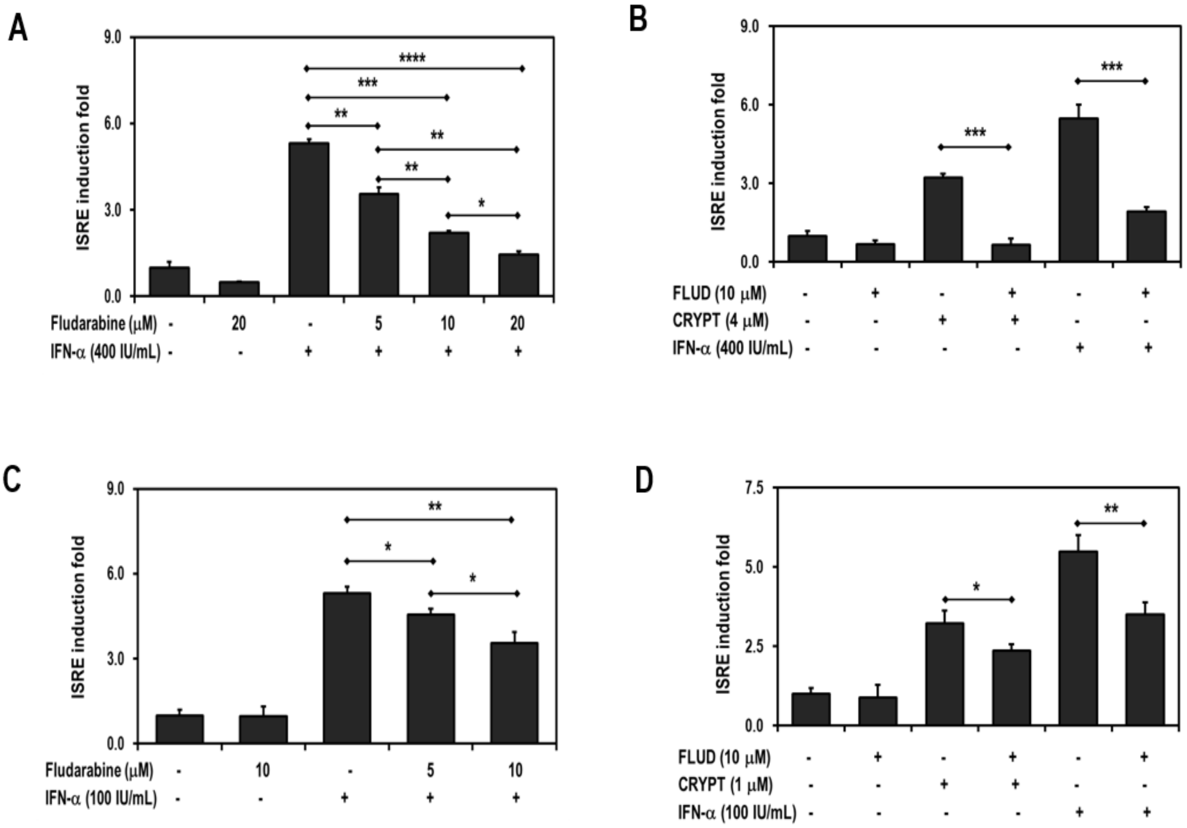

S 6: Activation of IFN-1 response by cryptolepine requires STAT1 in HepG2 and HEK293 cells. The HepG2 (**A** and **B**) and HEK293 (**C** and **D**) cells were cultured at 4×10^4 cells per well in a 96-well plate and transiently co-transfected as described under **S 1**. At 24 h post-transfection, the cells were pre-cultured with fludarabine for 2 h before culturing with IFN-α or cryptolepine and the ISRE induction was assessed after 24 h. Data are presented as the means, with the standard deviations as error bars, from three different experiments and each conducted in triplicate. *$p < 0.05$, **$p < 0.01$, ***$p < 0.001$ and ****$p < 0.0001$ (one-way ANOVA and Bonferroni's test; **A** and **C** or student's *t*-test; **B** and **D**).

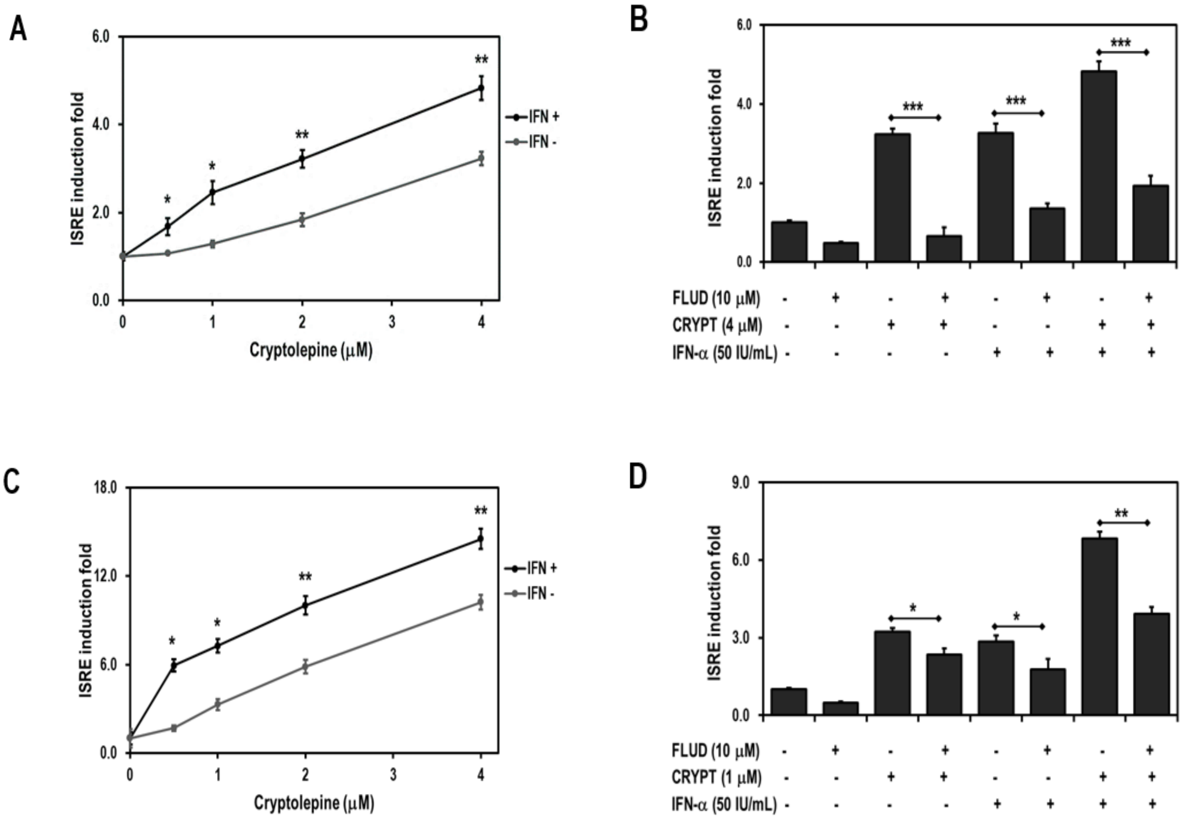

S 7: Cryptolepine enhances IFN-1 response activation by IFN-α in HepG2 and HEK293 cells. The HepG2 (**A** and **B**) and HEK293 (**C** and **D**) cells were cultured at 4×10^4 cells per well in a 96-well plate. After 24 h, the cells were transiently co-transfected as described under **S 1**. At 24 h post-transfection, cells were co-cultured with 50 IU/mL IFN-α and cryptolepine and the ISRE induction was assessed after 24 h (**A** and **C**). Next, the co-transfected cells were pre-cultured with fludarabine and co-cultured with IFN-α and cryptolepine and the ISRE induction was assessed after 24 h (**B and D**). Data are presented as the means, with the standard deviations as error bars, from three different experiments and each conducted in triplicate. $*p < 0.05$, $**p < 0.01$ and $***p < 0.001$ (student's *t*-test.)

S 8: Sequence of probes and primers of target genes

Gene	Primers and probes	Fluorophores
JAK1	Forward: 5’- CAATTGGCATGGAACCAACGAC-3’<br>Reverse: 5’-CAAATCATACTGTCCCTGAGCAAAC-3’<br>Probe: 5’-AGCAGTCAGTGTGGCGTCATTCTCC-3’	5’ FAM-3’ BHQ-1
TYK2	Forward: 5’-GAGATCCACCACTTTAAGAATGAGAG -3’<br>Reverse: 5’-GCTGTGCTGCCGGATATGC-3’<br>Probe: 5’- ACCTCTGTCACCTCGCTCTCCGCCA-3’	5’ FAM-3’ BHQ-1
STAT1	Forward: 5’GTTGCTGAATGTCACTGAACTTACC-3’<br>Reverse: 5’- AGCTGATCCAAGCAAGCATTGG-3’<br>Probe: 5’-CGCTCTGCTGTCTCCGCTTCCACTCC-3’	5’ FAM-3’ BHQ-1
STAT2	Forward: 5’-CCAGGTCACAGAGTTGCTACAG -3’<br>reverse: 5’-ACTTCCACAGTCAGTGACTCATTG -3’<br>Probe: 5’- CTCACCAGCAGCCTTGTTCGGACGG-3’	5’ FAM-3’ BHQ-1
IRF9	Forward: 5’-GGGAGCAGTCCATTCAGACATTG -3’<br>reverse: 5’-AGGTGAGCAGCAGTGAGTAGTC-3’<br>Probe: 5’- AGCAGCAGCAGCAGCAGCAGCC-3’	5’ FAM-3’ BHQ-1
OAS3	Forward: 5’-TCCGCCTGACATCCGTAGATC-3’<br>Reverse: 5’-TCCTCCGCAGCTCTGTGAAG-3’<br>Probe: 5’- AGCCTGGTGCCTGCCTTCAATGTCC-3’	5’ FAM-3’ BHQ-1
ACTB	Forward: 5’-TCACCCACACTGTGCCCATCTACGA-3’<br>Reverse:5’-CAGCGGAACCGCTCATTGCCAATGG-3’<br>Probe: 5’-ATGCCCCCCCCATGCCATCCTGCGT-3’	5’HEX-3’TAMRA

All the primers were designed to suit the following thermal cycling conditions: (i) an optimal melting temperature of 60°C; (ii) the difference in Tm of any two pairs of primers was not more than 2°C; (iii) the probes were designed to suit Tm of 68 – 70°C about 8 – 10°C higher than the Tm of primers.
